# Exploring trainer and trainee emotional talk in narratives about workplace-based feedback processes

**DOI:** 10.1007/s10459-017-9775-0

**Published:** 2017-04-29

**Authors:** A. A. Dennis, M. J. Foy, L. V. Monrouxe, C. E. Rees

**Affiliations:** 1 Centre for Medical Education, School of Medicine, University of Dundee, The Mackenzie Building, Kirsty Semple Way, Dundee, DD2 4BF Scotland, UK; 2 Chang Gung Medical Education Research Center, Chang Gung Memorial Hospital, Linkou, Taiwan; 3 Faculty of Medicine, Nursing and Health Sciences, Monash University, Melbourne, Australia

**Keywords:** Emotional talk, Workplace-based feedback, Trainer, Trainee, Narrative, Postgraduate medical education

## Abstract

Emotion characterises learners’ feedback experiences. While the failure-to-fail literature suggests that emotion may be important, little is known about the role of emotion for educators. Secondary analyses were therefore conducted on data exploring 110 trainers’ and trainees’ feedback experiences. Group and individual narrative interviews were conducted across three UK sites. We analysed 333 narratives for emotional talk using textual analysis: Linguistic Inquiry and Word Count. Furthermore, thematic framework analysis was conducted on the trainer narratives to explore aspects of feedback processes that are emotional. An additional in-depth little ‘d’ discourse analysis was conducted on selected trainer narratives to enable us to explore the complex relationship between the *whats* (reported events) and the *hows* (emotional talk). Trainer narratives did not differ significantly in positive or negative emotional talk from trainee narratives. By exploring the interplay of the *whats* and the *hows*, several aspects of feedback processes were identified as potentially emotional for trainers including trainers being concerned about upsetting learners and worried about patient safety. This was illustrated through numerous linguistic devices to establish emotional tone such as metaphoric talk and laughter. These findings suggest that feedback processes can be emotional for trainers. It highlights the need to better understand the ‘filter’ of emotion for trainers but also to better understand how emotion plays a role in feedback as a complex social process.

## Introduction

When learning, feedback plays a powerful role (Hattie and Temperly 2007). Within medical education, feedback aims to not only reduce the gap between current performance and future goals but also to ensure patient safety (Cleland et al. 2008; Challis et al. 1999; Hattie and Temperly 2007). Ensuring patient safety is of particular importance within a postgraduate context, where learners may be working independently with real patients in healthcare settings with guidance and support from more senior colleagues. There are multiple ways in which the literature, learners and educators conceptualise feedback (Urquhart et al. 2014). Some feedback scholars suggest feedback can only be considered as such if a change in behaviour or practice is evidenced (Boud and Molloy 2013). This definition of feedback arguably only refers to situations where an opportunity for feedback has been successful. Yet, if we want to truly understand why some feedback opportunities are successful while others are unsuccessful, we need to explore feedback processes in full. Therefore, in the current study we conceptualise and explore feedback in its many guises, focusing specifically on feedback as a process (Rizan et al. 2014). Researchers have been trying to identify and understand the factors that facilitate or hinder successful feedback processes (Eva et al. 2012; Sargeant et al. 2011; Teunissen et al. 2009; Urquhart et al. 2014; Watling and Lingard 2012). In particular, specific ‘filters’ have been identified that seem to influence this process such as motivations, expectations, and perceived instructor credibility (Eva et al. 2012; Sargeant et al. 2011). One filter that has been consistently flagged as particularly important is emotion (Eva et al. 2012; McConnell and Eva 2015; Värlander 2008; Urquhart et al. 2014).

### Emotion and feedback

The majority of empirical work on the relationship between emotion and feedback has explored the influence of emotion on how learners receive and process feedback from their teachers, conceptualising feedback as transmission. Eva et al. (2012, p. 23) assert that ‘receiving feedback is not an emotionally neutral task’. Feedback has the capacity to evoke emotions, which arguably may be why feedback can positively or negatively influence behaviour (Molloy et al. 2013). For example, when feedback is threatening to a person’s self-esteem, it may be less effective if the person focuses on threat-relevant information rather than the feedback per se (Hattie and Temperly 2007). Furthermore, valuable time may be spent processing negative emotions, delaying and reducing feedback utility, especially when the feedback is in conflict with learners’ self-perceptions (Bing-You and Trowbridge 2009; Overeem et al. 2009; Sargeant et al. 2007, 2009). Emotion influences many of our cognitive processes—including attention and memory (Levine and Edelstein 2009; Levine and Pizarro 2004)—both of which are important in the feedback process in situ and afterwards as individuals reflect on their feedback encounters (Urquhart et al. 2014; Levine and Edelstein 2009).

Despite the array of research exploring emotion and feedback from a learner perspective, to the best of our knowledge, no empirical research directly explores the impact of emotion on the feedback giver during such situations. If we consider the major impact that emotion plays on cognitive processing abilities, it seems reasonable to assume that emotion may influence feedback experiences from feedback givers’ perspectives too. Molloy et al. (2013) hypothesise that there are three key aspects of providing feedback that may be emotional for educators: (1) correctly making decisions about a student’s performance based on observational data; (2) having reservations about their own knowledge of the topic; and (3) being concerned about upsetting a learner. These key themes can be seen in some of the work on ‘failure to fail’, which highlights a reluctance from educators to fail learners within medicine (Cleland et al. 2008; Dudek et al. 2005; Rees et al. 2009; Watling et al. 2010). For example, Cleland et al. (2008, p. 804) cite that tutors ‘focused overwhelmingly on negative expected outcomes’ when faced with underperforming students, which played a role in their not wanting to report underperformance. When exploring the reported data many of the quotations demonstrate an emotional element, with some assessors implying that their emotions hampered them (Rees et al. 2009). Emotion comes through particularly when trainers describe giving negative feedback or failing a resident, ‘Now the problem with the “fail” … is that in this climate, many of us are very scared to do that’ (Watling et al. 2010, p. 1160). While these key articles provide many examples from feedback givers that are underscored by emotion, the issue of emotion is not explicitly discussed or explored in any depth.

### Narratives and emotion

One way to explore emotion is through narratives. Narratives comprise a sense making activity in which an individual not only describes an event, but also evaluates it, often incorporating emotional talk. Labov (1997) suggests that there are several component parts of a narrative: the abstract or summary; the orientation (time, place, and participants); the complicating action (sequence of events, turning point, the problem); the ‘most reportable event’ (the event that is least frequent but most impactful); the resolution; the evaluation; and the coda (returning to the present). Within a narrative, not all of these components may be included or may present in the order outlined here. Furthermore, narratives are ‘inextricably emotionally structured’ (Kleres 2011, p. 183) and provide an opportunity for individuals to further understand and share experiences, actions, identities, building and maintaining relationships, along with learning and teaching (Rees et al. 2013).

Previous work in the medical education literature has explored emotion through narratives, particularly in the context of professionalism (Rees et al. 2013; Monrouxe and Rees 2012; Monrouxe et al. 2014; Rees et al. 2015). Within these studies, textual and narrative analyses were utilised. For the textual analysis, Linguistic Inquiry and Word Count (LIWC; Tausczik and Pennebacker 2010) software was employed. This software identifies both positive and negative emotional talk and expresses the amount as a percentage within each narrative. Through LIWC analysis, patterns of emotional talk across narratives can be explored. Yet this analysis is limited in that emotion is not only expressed through the words a person uses but through other devices such as reported speech, repetition, hedges, judgments, metaphors, intensifiers, rhythm, stress and intonation, and laughter (Kleres 2011; Habermas et al. 2009; Rees et al. 2013). Little ‘d’ discourse analysis (Alvesson and Karrman 2000) allows for the consideration of these devices within the context of full narratives to understand the complex relationship between the *whats* (reported events) and the *hows* (e.g. emotional talk). By following a similar method to these professionalism studies, this paper aims to further understand the role of emotion in feedback experiences. To achieve this aim, this study analyses narratives from *both* trainers and trainees about their feedback experiences using LIWC, but then focuses in-depth on trainer narratives employing thematic and little ‘d’ discourse analyses.

### Research questions

1. To what extent do trainers and trainees employ emotional talk in their workplace-based feedback narratives?
2. Are there any differences between the trainer and trainee narratives in terms of the emotional talk employed?
3. What aspects of feedback appear to be emotional for trainers, as identified in their narratives?
4. How do trainers narrate feedback experiences with emotion and how does this relate to their description of the events?

## Method

### Study design

This study comprises a secondary analysis of data that originally explored the workplace-based assessment (WBA) and supervised learning event (SLE) experiences of trainees and trainers in the UK Foundation Programme (first two years post-graduation; Rees et al. 2014: see Box 1 for a glossary of terms). Here, we focus on the emotional talk within these workplace feedback experiences using a social constructionist epistemology, which asserts multiple ways of knowing and interpretations of reality (Crotty 2003). This constructionist epistemology aligned well with our desire to explore how participants interpreted and shared their experiences through personal incident narratives (PINs) in the context of a complex social process, namely feedback.

**Box 1:** Glossary of terms

UK Foundation Programme: Two year training programme between medical school and specialist/general practice training (Foundation Programme 2016) Workplace-based assessments (WBAs): ‘A system whereby doctors are assessed on clinical skills and other attributes in the context of his or her working environment’ (Saedon et al. 2012, p. 1) Supervised Learning Events: ‘An interaction between a junior doctor and trainer which leads to immediate feedback and reflective learning. They are designed to help junior doctors develop and improve their clinical and professional practice and to set targets for future achievements’ (Foundation Programme 2016, p. 13) Junior doctor: Junior doctor who is completing their training in the UK Foundation Programme. Educational/Clinical Supervisor: Doctors who are selected and trained to oversee a junior doctor’s clinical work and/or the foundations doctor’s educational progress during or across training placements (Foundation Programme 2016) Mini-Clinical Evaluation Exercise (Mini-CEX): ‘A workplace-based assessment tool… [involving] a focused, brief, observed, clinical encounter followed by feedback by the supervisor using a structured rating form’ (Weller et al. 2009, p. 633) Case-based Discussion (CBD): ‘The focus of case-based discussion… is the doctor’s clinical decision-making and reasoning’ (Brown et al. 2011, p. 85). In the context of the Foundation Programme, CBDs are junior doctors’ structured discussions of clinical cases in order to explore and give feedback on their clinical reasoning (Foundation Programme 2016) Direct Observation of Procedural Skills (DOPS): Assesses using a global rating scale focusing on 11 domains. (such as obtaining informed consent, aseptic technique, and overall ability to perform procedure) (Bould et al. 2009). In the Foundation Programme, the main aim of DOPS is to provide trainees with feedback on their interactions with patients while performing procedures (Foundation Programme 2016)	

### Sampling and recruitment

After ethics and institutional approval, data for the original study were collected at three sites across England, Scotland, and Wales (see Rees et al. 2014 for full details of sampling and recruitment). Maximum variation sampling, which seeks as wide a range of understandings and experiences as possible, was utilised (Kuper et al. 2008). First and second year junior doctors (JY1, JY2) and trainers (e.g., educational and clinical supervisors) across a range of settings (e.g., hospital and general practice) participated. Multiple methods of recruitment were used (e.g. email, notice boards, snowballing). Across the individual and group interviews, 110 individuals participated (34 JY1, 36 JY2, 40 trainers). Of the junior doctors, 31 were male (44%) and 39 female (56%) and the majority were under 30 years (n = 65; 93%). Of the trainers, 24 were male (60%) and 16 female (40%) and the majority were over 30 years (n = 37; 93%).

### Data collection

Fifty-five individual (34 with trainers; 21 with trainees) and 19 group (3 with trainers; 16 with trainees) interviews were conducted. The interviews explored participants’ understandings of SLEs and WBAs, and then used narrative interviewing techniques to elicit personal incident narratives of their workplace-based feedback experiences (see Rees et al. 2014 for further details). The interviews concluded when participants felt that their experiences had been fully explored. The interviews were audio-recorded and transcribed anonymously with paralinguistic information such as laughter also included in the transcripts. Participants also completed a personal details questionnaire asking for demographic and education-related information.

### Data analysis

In the initial study (see Rees et al. 2014 for more detail), we developed a coding framework through framework analysis in order to code the transcripts, (Ritchie and Spencer 1994) and employed Atlas-Ti to assist in data management. During the analysis process, the researchers also listened to the audio files to enhance our understanding of the data and the richness of the analysis. Framework analysis develops themes through a five-stage approach: Data familiarisation, thematic framework identification, indexing, charting, mapping and interpretation (Ritchie and Spencer 1994). Through this initial coding process, we identified narratives of SLE and WBAs that comprise the narratives analysed in this study.

For the purpose of secondary analysis in this study, SLE and WBA narratives were combined together as they comprised feedback using the same assessment tools i.e. the Mini Clinical Evaluation Exercise (Mini-CEX), Case-Based Discussion (CBD), and Direct Observation of Procedural Skills (DOPS). Furthermore, in our original study, we noted numerous similarities between SLEs and WBAs, with many of our participants not understanding the differences between the two other than a change in name, from WBA to SLE (Rees et al. 2014). Within the current study, complementary forms of analyses were used to explore the emotional talk used within the narratives: textual analysis (using LIWC) of all narratives, thematic framework analysis of trainer narratives, and in-depth little ‘d’ discourse analysis of selected trainer narratives.

#### Textual analyses using LIWC

In order to conduct the analysis, first, a validated text analysis tool called LIWC (Tausczik and Pennebacker 2010) was used to interrogate the narratives for both positive and negative emotional talk. LIWC calculates the percentage of words in any given text that are conceptually related to different emotions, ways of thought, and psychological states. Here we were interested in emotion. Examples of positive emotion words are ‘love,’ ‘nice,’ and ‘sweet.’ Negative emotional talk is split into three separate categories comprising anger (e.g. hate), anxiety (e.g. worried), and sadness (e.g. grief). MF prepared the narratives for analysis: she first removed any interviewer talk from the narratives; and second, followed the LIWC instructions to prepare the text files (Pennebaker et al. 2003, 2007). This included removing common fillers in language that are counted in the LIWC emotion language dictionaries such as ‘like’ and ‘well,’ and also removing any double negatives (e.g. ‘I was not uncomfortable’ became ‘I was comfortable’) so that they would be counted in the correct emotional context when analysed (see Monrouxe and Rees 2012 for further information about LIWC).

For this textual analysis, the qualitative data were quantified to highlight patterns within the data. This approach is not unusual for this type of analysis (Monrouxe and Rees 2012; Rees et al. 2015; Schiffrin 1994) and despite this, we still maintain an overarching qualitative approach underpinned by interpretivism, viewing reality as socially constructed and stressing the importance of context to language (Rees et al. 2013; Monrouxe and Rees 2012; Rees et al. 2015; Maxwell 2010). Descriptive statistics (i.e. frequencies) were first analysed, and then basic statistics were calculated, with tests run to establish whether the data were normally or non-normally distributed. P–P plots were drawn and skewness and kurtosis were examined for normality. The results of the tests indicated that the data were non-normal and therefore nonparametric tests were subsequently used to analyse the data (i.e. median and interquartile range, Mann–Whitney, and Wilcoxon signed-rank test).

#### Secondary thematic framework analysis

As part of this secondary analysis, we extended our coding framework from the original study (Rees et al. 2014) adding an additional theme with a number of underlying subthemes exploring our third research question: what aspects of feedback appear to be emotional for trainers, as identified in their narratives? To do this, MF, AD and CR went through a subset of trainer narratives to develop the initial framework. This process was both deductive (with the researchers using the three aspects that Molloy et al. (2013) identified as emotional) and inductive (with the researchers also exploring other aspects of providing feedback that could be emotional for trainers in the data). Then AD and CR both went through all of the trainer narratives using the extended framework to code the data independently with any disagreements being discussed and negotiated.

#### Discourse analysis

While LIWC is a helpful tool in establishing emotional talk in large amounts of qualitative data it lacks the ability to capture emotional tone established through other linguistic and paralinguistic means (Habermas et al. 2009; Kleres 2011; Monrouxe and Rees 2012; Monrouxe et al. 2014; Rees et al. 2015; Peterson and Biggs 2001). Therefore, in order to illustrate the rich and complex interplay between emotional talk and other, more subtle devices to establish emotional tone within the narratives, we present an in-depth little ‘d’ discourse analysis of four selected trainee narratives (Alvesson and Karrman 2000). We have chosen the narratives as they are fairly typical but represent diversity in terms of what trainers may find emotional about giving feedback. Indeed, together, they illustrate all eight of the sub-themes identified in our secondary thematic framework analysis. The narratives also feature a wide range of emotional talk and other linguistic devices to convey emotional tone such as pauses, hesitations, hedges, reported talk or thoughts, metaphoric talk and laughter. Furthermore, we choose four narratives rather than fewer in order to address common criticisms levelled at in-depth narrative analysis that insufficient examples are provided. Note that we focus on trainer narratives here in order to address the current gap in the literature for this stakeholder group.

## Results

From the 110 participants interviewed in the initial study, 333 narratives were identified and utilised for our secondary analyses in this study. 106 narratives (31.8%) were narrated by trainers, 146 (43.8%) by JY1, 77 (23.1%) by JY2, and 4 (1.2%) by JY where the training year was unspecified or unclear.

### Textual analyses

#### Overview of emotional talk within all the narratives

In terms of research question 1, 96% (n = 318) of the narratives contained emotional talk. Overall, there was more positive (*Mdn* = 1.58, IQ = .91–2.32) than negative emotional talk within the narratives (*Mdn* = 0.39; IQ = .00–.87; Z = −12.60, *p* < .001, *r* = −.69; large effect). However, 70% (n = 233) of narratives contained both negative *and* positive emotional talk. 23% (n = 75) of narratives contained only positive emotional talk and 3% (n = 10) contained only negative emotional talk. The most common positive emotional words that were included in the narratives were ‘good’ (n = 468), ‘useful’ (n = 127), and ‘well’ (n = 113). The most commonly used negative words were ‘difficult’ (n = 106), ‘problem’ (n = 55), and ‘bad’ (n = 47).

#### Differences in emotional talk between trainers and trainees

In terms of research question 2, while trainers (*Mdn* = 1.44, IQ = .98–2.27) had slightly less positive emotional talk than trainees (*Mdn* = 1.63, IQ = .90–2.33), this relationship was not statistically significant (Z = −.83, *p* > .05). Likewise, although the pattern was in the opposite direction for negative emotional talk, where trainer narratives (*Mdn* = .50, IQ = .20–.96) had more negative emotional talk than trainee narratives (*Mdn* = .36, IQ = .00–.82), this was also not statistically significant (Z = −1.55, *p* > .05).

### Thematic analysis

Within our extra theme: ‘Emotional aspects of feedback for trainers’, which extended our original coding framework (Rees et al. 2014), we identified eight sub-themes: Decision making, reservations about personal knowledge, upsetting learners, reflecting on own/others’ teaching, patient safety/experience, not wanting to fail, feedback resistance, and time restraints (see Table 1 for definitions of each of these subthemes and exemplar quotations).

**Table 1:** Results from thematic framework analysis

Emotional aspects of feedback for trainers	Exemplar quotations
1 Decision-making (modified from Molloy et al. 2013): This code refers to situations where trainers are concerned about correctly making decisions about a student’s performance based on observational data. This includes examples where trainers express concerns that they have insufficient information to make decisions about a student or they weigh up other information such as making allowances for trainees who do not want to pursue the specialty in which the feedback takes place	“So it was a very difficult one to assess that one because I know that the trainee can normally do better than that but it wasn’t really… you weren’t really looking at running a team meeting, it was how he handled the interruptions and all the rest of it which doesn’t normally happen…” (Female Doctor, Trainer 4) “I find it very difficult to grade people… from a brief interaction… you’re sampling a very small part of the medicine curricular as a whole and therefore it’s very difficult to say that they’re you know, that they’re going to be good at everything but actually from that little snapshot you get the impression that their… knowledge seems to be good” (Male Doctor, Trainer 17)
2 Reservations about personal knowledge (modified from Molloy et al. 2013): This code refers to situations where trainers have reservations about their own knowledge of a topic or skill or that someone else doubts their knowledge or skills	“I also did a doctor [in] difficulty course as well to try and help me to understand how to help her because… we’re never trained in these things” (Female Doctor, Trainer 3) “I don’t think that we’ve actually been sat down properly and told ‘right, this is the difference between this… this is to be formative, this is to be summative, this is how we would suggest that you conduct these and the problem with that… then is you are at risk of the trainee’s going ‘well such and such does it in this way which is different from that’ and it creates a little bit of tension” (Female Doctor, Trainer 21)
3 Upsetting learners (modified from Molloy et al. 2013): This code refers to situations where the trainer is concerned about upsetting the learner during or after feedback	**"**If the trainee made a complete hash of it I would probably give the feedback privately but providing it went well and the… trainee’s got reasonably broad shoulders I would give immediate feedback” (Male Doctor, Trainer 28) “you use those tools… try to pinpoint those things that they’re… concerned about [or whatever]… so it’s not been you know… why worry about what patients think or I’m worried about what the staff think, well let’s just not mind-read, let’s see what they [forms] say… and then you know it’s like ‘oh well look actually your mini-tab’s fine and everyone’s pretty happy with what you’re doing’… it depends on their level of security… we kind of used that as a way of sort of actually saying ‘everyone’s very… pleased with what you did’” (Male Doctor, Trainer 8)
4 Reflects on own and/or others’ Teaching: This code refers to situations where trainers are concerned that learners’ underperformance reflects badly on their own teaching performance or results from others’ poor teaching previously	“I thought that if… he’d done a bad job then you’d be thinking ‘oh my goodness, they’ve been taught incorrectly’” (Female Doctor, Trainer 21) “I asked the trainee to examine the hip and realised that he hadn’t really ever been shown since graduating how to examine a hip… Perhaps hadn’t really been shown or taught… to a terribly proficient level… as an undergraduate so I got him to examine the hip then I actually showed him what to do” (Female Doctor, Trainer 13)
5 Patient safety/patient experience: This code refers to situations where trainers raise concerns about patient safety or the patient experience more broadly during feedback encounters	“I stepped in once for surgical DOPS because the trainee was making a mess of it but I think that would happen if you’re standing watching anything [said with laughter] but that wasn’t particularly helpful because we knew the trainee was being heavily supervised” (Female Doctor, Trainer 3) “[It] puts you under pressure but… if it’s a poor performing junior then you need to spend a lot more time and support and find out why the performance is poor but also you know, you have to think about patients’ safety… if I thought that the junior was dangerous and things then I would… I would write an honest… ticket but because I knew from… watching her working with us for a couple of weeks I knew that, you know she needs a little bit more support and teaching and with that she was okay” (Female Doctor, Trainer 26)
6 Not wanting to fail: This refers to situations where trainers are concerned about the future adverse consequences of negative feedback they give to trainees. This sometimes manifests itself in trainers purposively not documenting poor performance on the required paperwork	“I was torn whether to write it up and that would look quite badly [laughs] on that junior so um in the end we said that we would leave it and treat it as… a bit of a blip in the performance” (Female Doctor, Trainer 26) “we then sort of said ‘you know actually I don’t want to put this down because I think it will sort of reflect badly on you as a trainee so why don’t we sort of work on that a little bit and then you can ask me again’… but she never came back [laughs] to me so I assume that she did find someone else” (Female Doctor, Trainer 11)
7 Feedback resistance: This refers to situations where trainers raise concerns about trainees’ resistance to feedback and/or their lack of insight into their own performance as part of feedback experiences	“Well we are talking about a supervisee who was very defensive about any failings and… that’s been the problems with the last two… that they have found it very hard to take on board anything which they perceive to be critical and… I think and have been very quick to place the blame for any shortcomings or difficulties they’ve had… [on others]” (Female Doctor, Trainer 39) “I think the hardest trainees I’ve ever had are the people who can’t reflect on what they’ve done. They don’t seem to have any ability to take a step out and actually view what’s going on and sometimes the only way that you can achieve that is by having very specific things to try and dissect and even then I think sometimes maybe it all comes down to personality and ability to be self-analytical, which some people don’t have I’m afraid” (Female Doctor, Trainer 5)
8 Time constraints: This refers to situations where the trainer feels that the trainee is taking up valuable time or trainers feel they have insufficient time to do assessments for trainees properly	“That took quite a long time. I think in the end we spoke for probably about twenty minutes on the one CBD for what was a fairly straightforward thing but he was determined to go into a tremendous amount of detail and you just feel a bit fatigued… and then when you realise there’s another one to do because he wanted to do two of them… [it was] a little wearing” (Male Doctor, Trainer 10) “It takes planning because generally when you do that you’re not seeing your own patients… so you have to block something off for it so that’s why [they are planned]” (Male Doctor, Trainer 8)

### Discourse analysis

We now present four trainer narratives that help us address our final research question: How do trainers narrate feedback experiences with emotion, and how does this relate to their description of the events? These illustrative narratives demonstrate how the linguistic features combine together and intersect with the ‘whats’. The emotion conveyed relates to various aspects of the feedback process identified through our thematic framework analysis such as patient safety/experience concerns. Importantly, the narratives highlight the complexity of emotion related to feedback as many demonstrate a mix of both positive and negative emotional talk. This is particularly noticeable in the transition from narrating the past event to evaluating the situation in the present. Please note that the narratives are emotive in nature and have some graphic elements that some readers may find emotionally confronting.

### Narrative 1: “I felt a bit disappointed that we perhaps failed her a bit…”

In this first narrative (see Box 2), Margaret (note that all names are pseudonyms), a female trainer, describes a CBD highlighting her surprise that the junior doctor [JD] ‘didn’t have a very good grasp on the case at all’. The interviewer then asks Margaret about the grade she gave the JD and what happened. At this point, Margaret utilises two metaphoric linguistic expressions, ‘I don’t think it *sparked* enough concern in me for me to have you know *raised the alarm.*’ In utilising the phrases *sparked* and *raising the alarm*, she likens her anxiety to that of being concerned of some danger. Yet although the JD had underperformed, she implies that she felt that patients were not necessarily in danger, and therefore did not need to escalate the situation further. Here we see how emotions interplay with two of our sub-themes identified in our thematic framework, specifically her decision-making around whether the trainee’s performance was of enough concern to escalate it further and her concerns about patient safety, which played a key role in her decision-making. This phrasing is also interspersed with laughter, perhaps as a form of coping when thinking about the unpleasantness of the experience (non-contextual) and/or concern about what the audience might think of her in the retelling (contextual).

**Box 2:** Margaret’s story (note that all names are pseudonyms)

Margaret: There’s only been one [struggling student] that I’ve done which was a case-based discussion [CBD] where she didn’t have a very good grasp of the case at all and yeah that was a bit of a surprise to me… Interviewer: Can you remember wha-what [grade] you gave her? And what happened? Margaret: I can’t actually um I mean I don’t think it sparked enough concern in me for me to have you know raised the alarm ((chuckle)) so to speak um but I can’t remember I gave her a very good grade yeah ((laughs with interviewer))… Interviewer: when you’re in that position, I mean how does that make you feel as a trainer? Margaret: um well I think it makes you feel a bit disappointed ‘cause she’d been on the ward for a while with us and you sort of question yourself about why she’s not improved more and I think a lot of the time the [junior] doctors are busy doing tasks but not really understanding fully why they’re doing them and… I think sometimes it makes you reflect on that ‘cause yo-you sort of surprised by their interpretation of what’s gone… so in that situation uh I felt a bit surprised, I felt a bit disappointed that we perhaps failed her a bit in not teaching her more thoroughly um but at least I felt that it had been identified and we did put a plan in place *(Margaret, Female Doctor, Trainer, CBD)*	

Margaret then goes on to discuss how the situation made her feel as a trainer. She highlights her ‘disappointment’ and ‘surprise’ multiple times throughout the narrative. She describes her concern that perhaps this JD’s poor performance reflects badly on the teaching that her and her colleagues are providing (a sub-theme identified in our thematic analysis above) saying, ‘*we perhaps failed her* a bit in not teaching her more thoroughly.’ Interestingly, she hedges this last statement (‘perhaps’), suggesting uncertainty about whether or not they had actually failed the JD. Throughout the narrative, Margaret uses mostly negative emotional talk but then summarises and evaluates the experience more positively where she says, ‘at least I felt that it had been identified and we did put a plan in place to try and improve her knowledge in that area.’ Here, she highlights that although the situation itself was unpleasant, a problem was identified and a plan was organised to resolve it.

### Narrative 2: ‘he was really quite rough and brutal and just… stabbing it in’

In this second narrative (see Box 3), John, a male trainer, describes his experience of conducting a DOPS assessment with a surgical trainee. He stops the trainee partway through his attempts at conducting a lumbar puncture on the patient because his approach is aggressive, as illustrated by John’s choice of negative emotional talk: ‘rough’ and ‘brutal’. Although John employs positive emotional talk to explain that the patient was ‘fine’ and not ‘really bother[ed]’ (because she was sufficiently anaesthetized), John communicates his horror at this junior doctor’s treatment of the patient, through his reported metaphoric talk, explaining that he ‘shudder[ed] to think’ how the doctor behaved (John implies worse) when he was not being observed as part of an assessment. Here, we can see clearly how John’s emotion interplays with one of our sub-themes described in our thematic framework analysis concerning patient safety/experience.

**Box 3:** John’s story

“In neurology we had a s- neurosurgical SHO who was working with us and um he was really quite rough and brutal and just going you know stabbing it [needle] in and I … just had to step in and say ‘stop, step back… I’m gonna take over’ and then we just finished the procedure and I discussed it with him afterwards that wasn’t really appropriate, patient was fine actually I think she’d had enough anaesthetic and didn’t really bother her but I, I didn’t give any opportunity to complete it I just thought ‘if he’s doing that, if he, if he’s thinks he’s behaving now and that’s how he’s doing it when someone’s watching then I shudder to think what he’s doing when no one’s watching’ um … I think he was a little disappointed um but… he acknowledged that he perhaps shouldn’t have done it like that… there was a number of problems with him because he was just being too brash and surgical with things … he wasn’t really taking notes properly on the ward round… but actually he did get better at the end… I wouldn’t have thought that this would work but it, he was a nice guy and becoming a bit more friendly with him and being friendly you know we went out few times after work and things like that it seemed to be that that fixed him because once he had a camaraderie with everyone… it was much easier to tell him ‘I want you do it like that’ and he would do it…” *(John, Male Doctor, Trainer, DOPS)*	

Afterwards, John then feeds back to the junior doctor that his attempts were inappropriate. Although he uses negative emotional talk (‘a little disappointed’) to describe the junior doctor’s reaction to his feedback, he explains that the junior doctor does acknowledge this feedback. John describes the junior doctor as ‘too brash and surgical’ and outlines other problems with his clinical practice such as sub-optimal note taking. John explains having to give him feedback on a number of occasions, and uses positive emotional talk saying he was a ‘nice’ guy who did ‘get better’ by the end of the rotation. John also attributes this getting better to his becoming more ‘friendly’ with him, explaining that ‘camaraderie’ made it ‘easier’ for John to give him constructive/developmental feedback and this ‘fixed him’. Some of these terms imply two conceptual metaphors for assessment relationships (Rees et al. 2009): assessment relationships as war and machine respectively. In terms of war, however, John implies that he is on the same side as this trainee (his comrade) rather than his enemy. In terms of machine, John implies that this trainee is a broken-down machine that John (the mechanic) has fixed. Taking altogether, John’s emotional and metaphoric talk illustrates clearly the emotional impact of this trainer-trainee relationship on John the trainer.

### Narrative 3: ‘this particular person didn’t much like being criticised’

In this third narrative (see Box 4), Tess, a nurse and trainer, discusses conducting a DOPS with a junior doctor (JD) and giving him feedback at the behest of the consultant. This narrative begins with Tess utilising negative emotional talk as she introduces the experience as quite ‘challenging’ and ‘concerning’ whilst she laughs, perhaps as a form of non-contextual coping. She mentions that the feedback was not *‘taken in the way I would’ve taken it’* (aligning with our feedback resistance sub-theme identified in our thematic framework analysis). She identifies herself as credible in giving the feedback, highlighting that she has years more experience in psychiatry than the JDs (which links with the sub-theme around concerns about personal knowledge). She then goes on to explain that it can be challenging for JDs to get used to the nurse-doctor relationship (‘whole nurse/doctor thing’). Laughingly, again perhaps for non-contextual coping, Tess then describes her view on how the JD perceives her, ‘*what’s this nurse doing feeding back to me that they don’t like the way I’m doing this.*’ Here, the pronouns are adversarial in nature utilising ‘me’ and ‘I’ in opposition to ‘this nurse’ and ‘they.’ In this statement she highlights that she believes that the trainee does not see her or her feedback as credible (again, linked to the sub-theme around reservations about personal knowledge).

**Box 4:** Tess’ story

Tess: I’ve had a recent example with an [JY1] um and a consultant actually asking me to assess this person taking a mental state from a patient and it being quite um… challenging ((laughs)) concerning and having to feed back on that first of all to the [JY1] and then to the consultant… [the feedback] wasn’t taken in the way I would’ve taken it but then we have years of experience on on the [JY1]s and [JY2]s in psychiatry um but it was quite difficult as well to give the feedback… I think the whole nurse/doctor thing takes a bit of getting used to… Interviewer: So is that partly an issue that maybe they’re perceiving you in a slightly Tess: as a ‘what’s this nurse doing feeding back to me that they don’t like the way I’m doing this’ ((laughs))… I actually spent quite a bit of time with the [JY1] and told them how I would’ve done it differently and wh-what it was about their interaction that I felt wasn’t as helpful as it could’ve been um and they were totally aware that I was gonna be feeding that back to the consultant you know um and it was fine actually it was I think initially the person concerned was a bit upset but it’s never nice is it? Giving constructive criticism ((laughs))… I think it’s different with different people some people accept it a lot easier than others and this particular person didn’t much like being… criticised although I didn’t feel like I was criticising *(Tess, Female Nurse, Trainer, DOPS)*	

She then describes spending quite a lot of time with the trainee (linked with the sub-theme ‘time restraints’ from our thematic framework analysis), explaining what she felt the trainee could have done differently. Tess then evaluates the situation more positively saying, ‘it was fine actually.’ She summarises the situation saying that ‘initially the person concerned was a bit upset’ suggesting that the trainee got over their initial upset. She then reflects on her discomfort with giving trainees negative feedback, ‘*but it’s never nice is it, giving constructive criticism?*’ again laughing as she does so, still suggesting contextual coping. Here, her emotion interplays with her concerns about upsetting the learner (another sub-theme identified in our thematic framework).

### Narrative 4: ‘at least she has insight and so it’s not a disaster’

In this final narrative (see Box 5), Jennifer, a female trainer, discusses facilitating a CBD about the medical management of heart failure with a foundation trainee destined for a surgical career. She explains that the trainee performs sub-optimally, describing her as not performing as well as she ‘should’ or ‘could’ and using positive emotion talk coupled with negatives ‘wasn’t as good’, and sometimes with hedges such as ‘maybe’ and ‘quite’. While she is clearly underwhelmed by the trainee’s performance, she classifies it as ‘satisfactory’, ‘probably okay’ and with laughter, ‘alright for someone who wants to be a surgeon’, illustrating the challenges around her decision-making regarding this trainee (one of our sub-themes identified in our thematic framework analysis). Jennifer also uses negative emotion talk coupled with negatives and sometimes hedges to justify why she did not fail the trainee (e.g. ‘it wasn’t a disaster’, ‘it’s not a disaster’, ‘I don’t think that anything had been dangerous’). Again, we can see how emotion interplays with her concerns about patient safety and challenging decision-making (again sub-themes identified in our framework).

**Box 5:** Jennifer’s story

Jennifer: I think it was a case-based discussion…she [trainee] wasn’t as well prepared as she *could* have been it wasn’t as good as it *could* have been but she was about to move on, and it wasn’t a disaster and so it wasn’t worth like not passing her… So I didn’t give her *good* marks I just said that she was you know ‘satisfactory’ or whatever because it was probably okay… it just wasn’t maybe as good as it *should* have been or it *could* have been
Interviewer: Yeah did you give her that that feedback…?
Jennifer: Yeah and we did it in a kind of informal slightly jokey way she… she actually said to me ‘oh, maybe I should have been a bit more’ I don’t know I can’t remember what she actually pointed out and I said ‘yeah, well’, I *agreed with* her self-assessment and I think perhaps because she had that self-assessment that she *knew* that it hadn’t been quite as good that made me think ‘well at least she has insight and so it’s not a disaster’… Plus… I thought ‘she’s probably alright ((says laughingly)) for someone who wants to be a surgeon’… because she’s never gonna have to do this… in the future… and I don’t think that anything had been dangerous… and it was more a lack of preparation on her part *for* the assessment rather than anything particularly dangerous that she’d done… and like I say… in four months’ time not even that’ cause it was towards the end of the year she was never gonna have to do this again anyway so it seemed ridiculous to fail her for…
Interviewer: how does that make you feel as a trainer?
Jennifer: It depends *why* they’re not performing if it’s ‘cause they’re being lazy or unmotivated it’s frustrating’ cause you’ve got so many other things you wanna do or you could be doing with your time… you’re sitting there… with them for 20 min and they’ve not prepared that is frustrating… If it’s because they don’t know… you’re more willing to put in time because you think ‘okay here is an actual area of deficiency, we need to work on this this particular issue’…. it was [frustrating] because she was such a smart person… but obviously just focused on heading for a surgical life… and not that bothered about heart failure… *(Jennifer, Female Doctor, Trainer, CBD)*

Additional reasons given for her not failing the trainee included that ‘she wasn’t as well prepared as she could have been’, ‘she had insight (into her performance)’, ‘she was a smart person’, ‘she was about to move on’, ‘she was never gonna have to do this again’, and Jennifer’s over-riding belief that it would have been ‘ridiculous’ to fail her (aligning with our ‘not wanting to fail’ sub-theme identified in our thematic framework analysis). Jennifer finishes her narrative by talking about our final theme identified in our framework: time constraints. She highlights that trainees that are ‘lazy’, ‘unmotivated’ or ‘not prepared’ can be frustrating ‘cause you’ve got so many other things you wanna do or you could be doing with your time’. She contrasts this with trainees who ‘don’t know’, suggesting she is ‘more willing to put the time in’. She is clearly frustrated by this specific trainee because she was ‘smart’ but ‘not bothered about heart failure’ because of her chosen surgical career path implying that the trainee was wasting her time with her ‘lack of preparation.’

## Discussion

Although previous literature has hinted at emotion playing a role in feedback from feedback givers’ perspectives (Cleland et al. 2008; Dudek et al. 2005; Molloy et al. 2013; Rees et al. 2009; Watling et al. 2010), to our knowledge, this is the first empirical study to explore this issue explicitly. We examined the emotional talk utilised in feedback narratives with a particular focus on trainer narratives to address the gap in the literature. Using LIWC we found that the majority of trainer and trainee feedback narratives contained mostly positive *and* negative emotional talk. Trainer narratives did not differ significantly from trainee narratives in terms of the proportion of positive and negative emotional talk. In addition to exploring the quantity of emotional talk, we considered particular aspects of the feedback process that were emotional for trainers. Here, we found a range of issues including trainers’ concerns around their decision-making, reservations about their personal knowledge, fears about upsetting learners, worries that student underperformance reflected badly on their own teaching, trainers not wanting to fail trainees, trainees’ resistance to feedback, time restraints, and patient safety concerns. We also describe how these issues interplayed with various linguistic and paralinguistic features to establish emotional tone such as metaphoric talk, laughter, pauses, hedges and negatives. Indeed, the narratives highlight the complexity of emotional talk where both positive and negative emotional talk can be interwoven particularly in the transition between relaying the story to evaluating it in the present moment.

## Comparisons with existing literature

Our findings extend existing literature around emotion-as-a-filter by demonstrating that feedback interplays with emotion for trainers as well as trainees (Eva et al. 2012; Urquhart et al. 2014; Värlander 2008). This aligns with broader literature on emotion and cognition, asserting that emotion guides processes such as attention and memory, impacting both trainers and trainees (Levine and Edelstein 2009; Levine and Pizarro 2004).

The analyses employed have highlighted the range of linguistic devices within participants’ narratives (e.g., metaphors, repetition, laughter) used to portray emotion (Habermas et al. 2009; Kleres 2011; Rees et al. 2013; Monrouxe and Rees 2012). By exploring the relationship between the *‘whats’* and *‘hows’*, our study provides us with further insights into key aspects about feedback experiences that have an emotional component for trainers. In particular, it provides evidence regarding emotional aspects for trainers such as concerns around correct decision-making based on observational data, about their own knowledge and about upsetting learners (Molloy et al. 2013). Additionally, our findings extend existing literature by highlighting five further aspects that are emotional for trainers: concerns that underperformance reflects poorly on their own teaching, patient safety issues that arise during feedback encounters, time constraints, trainers not wanting to fail trainees, and feedback resistance. Some of these issues have been previously raised in assessment research at the undergraduate level. For instance, Cleland et al. (2008) stated that one of the factors impacting on ‘failure to fail’ relates to tutor self-efficacy. They found tutors, especially less experienced tutors, were more likely to feel that a learner’s underperformance reflected on them as teachers. Feedback resistance was also identified as particularly challenging for trainers, aligning with work on remediation. For example, Guerrasio et al. (2014) suggest that individuals who are perceived as trying and having insight, even if they had other areas of inadequate performance, may be more likely to be tolerated and supported by trainers. Cleland et al. (2013) reviewed the literature on remediation, which suggests that individuals who were closed to feedback and could not self-assess pose significant challenges to trainers.

Furthermore, time constraints provide challenges to trainers linking to decision making. Rees and Shepherd (2005) found that both undergraduate students and assessors highlighted that if an assessor had spent only a short period of time with a student, it would increase the difficulty of making judgements about performance. This finding also aligns with broader issues about balancing service and training conflicts which has been identified as a key priority issue for future medical education research (Dennis et al. 2014). This in turn connects with our patient safety and experience theme where trainers are concerned about the patient whilst trying to balance training needs. In particular, patient safety is a clear consideration for trainees, with both trainees and trainers having a duty of care to act if they believe patient safety is at risk (General Medical Council 2012). This responsibility is something that is central to the feedback event, particularly in a postgraduate context where the learner is acting as a healthcare professional. Indeed, one might argue that the stakes are higher in this postgraduate context. As Cleland et al. (2008) suggest, without good feedback, continuing underperformance could lead to a learner ultimately becoming an incompetent or unsafe doctor. It therefore makes sense that patient safety issues during a feedback encounter could be highly emotional for trainers.

### Methodological strengths and limitations

As with any study, our work has limitations. One important limitation is that we may have over-estimated the emotionality of trainer feedback experiences. For example, we know that memorable events often have an emotional component (McConnell and Eva 2015). Therefore, participants are more likely to narrate emotional events than less emotional events that have either been forgotten or are less interesting. This may be particularly important in the case of trainers, where they could have experienced hundreds of workplace-based feedback events in their clinical teaching roles but may only remember an emotional few. Second, it is important to note that narratives are a re-telling of an event and therefore the emotional talk used in the description of the event may not necessarily fully represent the emotions experienced in the moment. Finally, it is important to note that the inferential statistics presented on the LIWC data need to be interpreted with caution as quantitative data were neither wholly independent nor matched.

Despite these challenges, there are a number of methodological strengths in this study. Firstly, the study accessed a large sample of narratives collected from a variety of individuals at multiple sites. Second, we have focused our paper on the perspective of trainers, which addresses a clear gap in the research literature. Exploring only the learner perspective has been highlighted as a key weakness in the feedback literature recently (Urquhart et al. 2014). This paper adds the trainer perspective to the conversation. Finally, the study has also included complementary forms of analysis. It has not only considered the ‘whats’ through the LIWC textual analysis but it has also explored the complex interaction between these ‘whats’ and ‘hows’ through the thematic framework and little ‘d’ discourse analyses, providing a more complete picture of how emotion plays a role in feedback events.

### Implications for educational practice and future research

Identifying that feedback experiences can be emotional for both the trainer and trainee has educational implications. By ignoring and avoiding emotion, we are denying an important influence on feedback processes. For instance, emotion may be playing a large role in the ‘failure to fail’ phenomena in medical education. Therefore, faculty development in particular should support trainers to reflect on the emotional aspects of the feedback process. For example, the narratives presented in this paper could be used as trigger materials in faculty development sessions on feedback to encourage reflection on how feedback practices are imbued with emotion.

In terms of further research, additional studies need to explore the impact of emotion on the feedback practices of trainers, in addition to understanding the multi-dimensional aspects influencing those feedback practices, particularly in the postgraduate learning environment where issues like patient safety add additional complexity. Furthermore it would be important to explore issues such as how does an individual’s perception of their role as an educator influence their emotional connection with the processes of feedback or people involved in those processes. It would also be invaluable to capture feedback events in situ to explore emotional talk during actual feedback events, enabling us to make comparisons between the events themselves and the ways in which trainers and trainees reflect on the events afterwards. While this research has been done in an undergraduate context (e.g. Urquhart 2015), it is still yet to be explored fully in a postgraduate context. This particularly relates to Boud and Molloy’s (2013) critique that feedback can only be considered feedback if there is an impact on practice. This could be explored from both trainer and trainee perspectives, where arguably both parties can ‘learn’ from feedback events. Related to this point, we need to explore the impact of emotion not only on an individual level but also on a social level sensitive to context. Feedback is not a solo activity: it is a multifaceted and dynamic social process occurring in context (Ajjawi 2012; Ajjawi et al. 2017). Urquhart et al. (2014) highlight that we need more research where we consider everyone involved in the feedback process to further appreciate this dynamic. If we consider feedback like an intricate dance (Monrouxe et al. 2009), we need to understand emotion not only as an individual factor but consider how it influences the rich interactions in that dance. For instance, does emotion influence the relationships between the individuals? How does the emotion of the event impact future feedback events between the two individuals or other individuals? Ultimately, through understanding how emotion influences feedback we might find better ways to enhance its success.
